# SEOM Clinical Guideline for bone metastases from solid tumours (2016)

**DOI:** 10.1007/s12094-016-1590-1

**Published:** 2016-11-28

**Authors:** C. Grávalos, C. Rodríguez, A. Sabino, M. Á. Seguí, J. A. Virizuela, A. Carmona, J. Cassinello, D. Isla, C. Jara, M. Martín

**Affiliations:** 1 Medical Oncology Department, Hospital Universitario 12 de Octubre, Madrid, Spain; 2 Hospital Universitario de Salamanca, Salamanca, Spain; 3 Grupo ONCOAVANZE, Seville, Spain; 4 Corporació Sanitària Parc Taulì, Sabadell, Spain; 5 Complejo Hospitalario Regional Virgen Macarena, Seville, Spain; 6 Hospital Universitario J.M. Morales Meseguer, Murcia, Spain; 7 Hospital Universitario de Guadalajara, Guadalajara, Spain; 8 Hospital Clínico Universitario Lozano Blesa, Saragossa, Spain; 9 Hospital Universitario Fundación Alcorcón, Madrid, Spain; 10 Hospital Universitario Gregorio Marañón, Madrid, Spain

**Keywords:** Biphosphonates, Bone metastases, Denosumab, Skeletal-related events (SREs), Radium 223, Zoledronic acid

## Abstract

Bone metastases are common in many advanced solid tumours, being breast, prostate, thyroid, lung, and renal cancer the most prevalent. Bone metastases can produce skeletal-related events (SREs), defined as pathological fracture, spinal cord compression, need of bone irradiation or need of bone surgery, and hypercalcaemia. Patients with bone metastases experience pain, functional impairment and have a negative impact on their quality of life. Several imaging techniques are available for diagnosis of this disease. Bone-targeted therapies include zoledronic acid, a potent biphosfonate, and denosumab, an anti-RANKL monoclonal antibody. Both reduce the risk and/or delay the development of SREs in several types of tumours. Radium 233, an alpha-particle emitter, increases overall survival in patients with bone metastases from resistant castration prostate cancer. Multidisciplinary approach is essential and bone surgery and radiotherapy should be integrated in the treatment of bone metastases when necessary. This SEOM Guideline reviews bone metastases pathogenesis, clinical presentations, lab tests, imaging techniques for diagnosis and response assessment, bone-targeted agents, and local therapies, as radiation and surgery, and establishes recommendations for the management of patients with metastases to bone.

## Introduction

Patients with solid tumours are highly susceptible to develop bone metastases. While any malignancy may metastasize to bone, it is most prevalent in advanced breast (70–80%), prostate (70–80%), thyroid (60%), lung (10–50%), and renal cancers (30%) [1–3]. Incidence of bone metastases is also increasing in other cancers, probably owing to improved tumour control at other disease sites. Proximal femur, pelvis, vertebrae, and skull are frequent locations, being metastases in distal bones rare [4].

Bone metastasis is a devastating condition that can have a negative impact on the lives of patients with advanced cancer in many ways. They are also associated with significant consumption of healthcare resources that generate a substantial economic burden for the Healthcare System [5].

Normal bone formation is a coordinated dynamic process of active bone production by osteoblasts and bone remodeling and resorption by osteoclasts. This fine balance is mediated by a variety of local and systemic factors, such as transforming growth factor-beta (TGF-β), insulin growth factor (IGF), bone morphogenic protein, platelet-derived growth factor (PDGF), prostaglandins, and parathyroid hormone, as well as receptor activator of nuclear factor kappa-B ligand (RANK-L), a member of tumour necrosis factor (TNF) family, that is a key factor for osteoclast production.

When cancer metastasizes to bone, deregulated bone remodeling occurs. Metastasizing tumour cells mobilize and sculpt the bone microenvironment to enhance tumour growth and to promote bone invasion. Bone metastases disrupt this complex interplay through an organized and multistep process involving tumour intravasation, cell survival in the circulatory system, extravasation into surrounding tissue, initiation and maintenance of growth, vascularization, and angiogenesis. Tumour invasion into bone is associated with osteoclast and osteoblast recruitment, resulting in the liberation of growth factors from the bone matrix, which can feed back to enhance tumour growth resulting in the ‘vicious cycle’ of bone metastasis [6].

## Clinical and laboratory manifestations of bone metastases

Pain is the most common symptom of bone metastases. It is usually focal, well located, and associated with functional impairment, and may appear before imaging evidence of the disease.

Pathological fracture, spinal cord compression, need of bone irradiation, and need of bone surgery, usually to correct fractures or spinal deformities, are bone complications gathered in the category of skeletal-related events (SREs). Hypercalcaemia is not considered as a SRE in clinical trials, because it is easily reversible and can be a paraneoplasic syndrome in the absence of bone metastases. The development of an SRE determines poor prognosis (impact in quantity of life) [7] and a higher probability of a new bone event [impact in quality of life (QOL)].

### Laboratory tests

Elevated levels of bone turnover markers are proportional to the extent of skeletal involvement in patients with bone metastases [8]. Bone alkaline phosphatase, an isoform of alkaline phosphatase, is a relatively specific indicator of osteogenesis and shows a good correlation with the presence and spread of bone metastases, mainly in breast and prostate cancer, although its clinical application is limited by its relatively low specificity [9]. Urinary markers telopeptides, N-terminal (NTx) and C-terminal (CTx), are bone breakdown products of type I collagen released during the bone resorption. Risk of skeletal complications and disease progression is duplicated when NTx levels are moderate/high [10] and normalization of NTx and CTx excretion rates is associated with relief of symptoms and reduced incidence of SREs [11]. Bone turnover markers may be helpful in monitoring the efficacy of bisphosphonates (BPs). However, changes in urinary levels of NTx and CTx require long periods of time [12] and their use in the routine care is still controversial.

MiRNA, small non-coding RNA molecules, regulate gene expression and can be detected in early stages of bone invasion [13]. Several miRNAs (miR-10b, miR-16, and miR-378) have been proposed for the diagnosis of bone metastases in patients with breast cancer with acceptable sensitivity (64%) and specificity (69%), and miR-326 has been proposed as a biomarker for monitoring metastatic progression in bones [14].

## Bone metastasis imaging

Bone metastases imaging is essential for tumour staging and for monitoring the therapeutic response. Bone metastases are typically classified as lytic, sclerotic, or mixed. Lytic metastases prevail in the absence of bone production, being typical of myeloma, kidney cancer, and melanoma. On the contrary, metastases acquire sclerotic patterns wherever the osteoblastic activity dominates, as in prostate cancer. Often, lesions show a mixed appearance, as it can happen in breast or lung cancer. The differential diagnosis with benign processes can be complex, especially in the elderly.

### Diagnosis

Plain X-rays are frequently used, but their sensitivity is low.

Concerning nuclear medicine, two imaging methods applying different physical principles and reconstruction procedures are available: scintigraphy/single photon emission computed tomography (SPECT) and positron emission tomography (PET). Both allow hybrid image systems by combining them with computed tomography (CT) or magnetic resonance imaging (MRI): SPECT/CT, PET/CT, and PET/MRI. Tracers used within these techniques can be distinguished according to their functional mechanism, by exploiting different pathophysiological principles. Radionuclide bone scans (bone scintigraphy and SPECT) make use of ^99m^Technetium (^99m^Tc) diphosphonate as a tracer, which is bone matrix-specific, being the deposit proportional to blood flow and the osteoblastic activity [15]. This tracer identifies the bone reaction to the tumour, but is unable to detect the bone-marrow involvement [16]. As a result, ^99m^Tc-based imaging does not reliably distinguish between reparative osteoblastic activities (flare) versus (vs) true disease progression. Other techniques are generally required for solitary lesions or equivocal scintigraphy findings. Fused SPECT-CT may yield lower false-positive rates, since it allows morphological correlations [17, 18].

Other techniques, such as 18-fluoro-2-deoxy-d-glucose ([18] F-FDG) PET or MRI, can detect metastases prior to the onset of the osteoblastic activity. Compared to SPECT [18], F-FDG PET has a greater spatial resolution and allows the quantification of maximum standard uptake values (mSUV), which discriminates between malignant and benign lesions. Unlike bone scintigraphy, it is assumed that [18] F-FDG uptake is a specific marker for tumour glycolysis, but does not assess the microenvironment status. In this context, [18] FDG-PET has shown higher sensitivity than bone scintigraphy (93 vs 81%) [19]. The reduction in false-positive rates may be due to both intrinsic tracers’ mechanisms and the fusion with CT imaging. On the other hand, the sensitivity of PET/CT is higher for lytic tumours in comparison with blastic lesions; several factors may be involved, being one of them the greater biological aggressiveness of the former [20]. Accuracy values will also depend on the tumour and histological subtypes. For example, sensitivity is also lower in certain tumours, such as prostate cancer, in which [11] C-choline PET may yield better results.

MRI is the technique of choice whenever a precise anatomical delineation is necessary. Morphologic MRI is superior to radiographs or scintigraphy in the assessment of focal lesions, since it allows the detection of bone-marrow replacement by tumour cells before the onset of trabecular changes. Since specificity can be lower to [18] F-FDG PET/CT, MRI studies should not be read isolated, but integrated with other imaging modalities [16]. New functional techniques, such as whole-body diffusion-weighted (DW) or dynamic contrast-enhanced (DCE) MRIs, are beginning to be explored with exciting possibilities [21].

It is important to prove the diagnosis of metastases by biopsy in impending or complete pathologic fracture in a patient with solitary lesion, because infections and primary sarcomas can have a similar appearance. If a patient has a history of cancer without prior documentation of bone metastases, TC-guided fine needle aspiration and core biopsy are excellent method to document the presence of metastatic disease.

### Response assessment

Bone involvement is the only one that contemplates specific measurability criteria with regard to tumour response assessment [22]. Conventional radiographies are insensitive and do not allow early evaluations [23]. One of the issues is that tumour-induced osteoblastic activity is indistinguishable from reparative osteoblastic changes. As a result, bone scintigraphy assessing the number and intensity of hotspots has shown low performance in this situation. Ongoing sclerosis may constitute a sign of response. However, in approximately 50% of responders, no changes in bone scans could be observed, while in around 40% of non-responders, progression was not apparent for 6–8 months, in an early study [24]. A transient morphologic worsening by inflammatory and reparative phenomena of lesions that are actually responding is sometimes referred to as “flare”. This is a limitation for the use of bone scintigraphy in clinical practice or as endpoint in clinical trials, especially when the lytic component dominates.

PET/CT can predict responses earlier [25], but at present, evidence is still lacking to recommend the routine use. Metastases in progression generally become more avid to [18] F-FDG. Likewise, an increase in sclerosis of the lesions over time without mSUV increases is usually associated with true responses, although it does not appear that this is always the case [16]. The Positron Emission Tomography Response Criteria in Solid Tumours (PERCIST) intend to homogenize the standards of evaluation by this method [26]. Studies are ongoing to determine the value of other promising techniques, such as [18] F-NaF PET (NCT00882609).

On the other hand, morphologic MRI seems to be useful to detect tumour progression but not so much to predict early response [27]. DW-MRI changes or multiparametric approaches may be helpful, but more data are warranted due to the great heterogeneity in metastatic patterns and behaviors.

In summary, there are currently new techniques with enhanced sensitivity and specificity for staging the skeleton and evaluating the response, although more experience is required before generalizing their use. For screening purposes, ^99m^TC, SPECT/CT, or [18] F-FDG-PET/CT is usually recommended, depending on the type of tumour, histology, and clinical setting. MRI confirmation is suggested for equivocal or solitary lesions. Whole-body MRI with T1, T2, or DWI-weighted sequences has shown promising results, but further evaluation is still recommended. Hybrid imaging techniques (e.g., SPECT/CT or PET/CT) may yield higher sensitivity and improved specificity. Some data indicate that [18] F-FDG PET/CT can be a good method for early response assessment. In the future, new techniques of fusion, integrating new tracers, surely will enable us to take another step forward.

## Medical treatment options for bone metastases

Appropriate management of bone metastases is essential, since about 40% of patients with bone metastases will develop a bone event in the absence of preventive therapy [28].

### Biphosphonates

Biphosphonates structure is similar to endogen pyrophosphate. Therefore, they are able to bind to mineralized bone matrix in areas of high bone turnover and potently inhibit of osteoclast-mediated bone resorption.

Zoledronate or zoledronic acid (ZA), the most effective third-generation bisphosphonate in preventing SRE, is administered at a dose of 4 mg intravenous (iv) during 15 min every 3 (q3w) or 4 weeks (q4w), with supplemental calcium and vitamin D. So far, the best dosing interval is still to be determined [29]. Zoledronic acid has Europe and United States regulatory approval for prevention of SREs. It is recommended to start at the bone metastases diagnosis, even the patient is asymptomatic, and should be continued indefinitely during the disease [30].

The most relevant adverse event of zoledronate is nephrotoxicity, a limitation for its use in cancer patients receiving cisplatin therapy, and particularly for lung cancer patients that are associated with comorbidities, older age, and tobacco use. Its administration needs renal function monitoring and dose adjustments. Acute-phase reactions (chills, fever, bone pain, and fatigue), hypocalcaemia, and osteonecrosis of jaw (ONJ) are other side effects. Risk of ONJ increases if previous dental trauma, infection, or surgery and with long-term administration of the drug.

#### Breast cancer

Two studies were conducted in patients with metastatic breast cancer (MBC). The first study included also multiple myeloma and was designed as a non-inferiority trial comparing zoledronic acid and pamidronate [31]. Although there were no differences across the entire study population in the proportion of patients with a SRE (43 vs 45%)—primary endpoint—among patients who had breast carcinoma with at least one osteolytic lesion, the proportion with an SRE was lower with zoledronate compared with pamidronate (48 vs 58%), but without statistical significance (*p* = 0.058). The median time to first SRE was significantly longer with ZA (310 vs 174 days, *p* = 0.013). In the second study in 228 Japanese women with bone metastases, zoledronic acid at doses of 4 mg iv q4w over a year reduced the risk of SREs by 39% compared to placebo (RR 0.61; *p* = 0.027) and the rate of patients experiencing at least one SRE, compared to placebo (29.8 vs 49.6%, *p* = 0.003), significantly delaying the onset of the first SRE [32].

Recently, two randomized trials have evaluated the administration of zoledronic every 12 weeks instead 4-week interval. In the OPTIMIZE 2 [33], female patients with bone metastases from MBC who previously received ≥9 doses of iv BPs (zoledronic or pamidronate) during the first 10–15 months of therapy were randomized (1:1) to receive zoledronate 4 mg iv q4w or q12w. The primary endpoint was the proportion of women with ≥1 SRE on study. The SRE rate was 22 and 23.2% in the zoledronic q4w and q12w arms, respectively. In the second trial, Himelstein et al. [34] compared during 24 months zoledronic q4w and q12w from the first dose of the drug. A total of 1822 patients with MBC (*n* = 833), myeloma, and other solid tumours were randomized. The proportion of SRE was 29.5 vs 28.6% (*p* = 0.79) for monthly and every 3 months, respectively, showing that zoledronic administered every 3 months is non-inferior to zoledronic administered monthly for 24 months.

In summary, zoledronic acid reduces SREs incidence in MBC patients with lytic bone metastases. Recent results support the dose of 4 mg every 12 weeks in patients who have received at least 1 year of q3w zoledronic and also this option could be considered in MBC from the beginning of the treatment.

#### Prostate cancer

About 90% of castration-resistant prostate cancer (CRPC) patients present imaging evidence of bone metastatic disease [35]. Zoledronic acid, denosumab, and radium-223 are the approved bone-targeted agents for bone metastases from this disease [36].

In a randomized, double blind, placebo-controlled, phase III trial, including 643 patients with bone metastases from CRPC, 4 mg zoledronic acid decreased incidence of SREs (33.2 vs 44.2%, *p* = 0.021) and increased the median time to the first SRE (488 vs 321 days, *p* = 0.01) when compared to placebo [37]. No differences in overall survival (OS), progression free survival (PFS) or QOL were observed between the groups of treatment. With a 24-month follow-up, zoledronic acid reduced SRE risk in 36% (RR 0.64; *p* = 0.002) and bone pain.

In contrast, zoledronic acid has not demonstrated any benefit in castration-sensitive prostate cancer. The phase III CALGB 90202 trial (Zoledronate in Preventing Skeletal [Bone]-Related Events in Men Who Are Receiving Androgen Deprivation Therapy For Prostate Cancer and Bone Metastases) [38] was early interrupted since no statistically significant differences between ZA and placebo were observed in the time to first SRE, primary endpoint, after 645 men recruited and 299 SREs observed (31.9 months in ZA vs 29.8 months in placebo; HR 0.97). Overall survival was similar (HR 0.88; 95% CI 0.70–1.12). Therefore, zoledronic acid is not approved for patients with bone metastases from castration-sensitive prostate cancer.

In summary, zoledronic acid is the only iv biphosphonate approved for SREs prevention for CRPC. However, it is not approved for patients with bone metastases from castration-sensitive prostate cancer.

#### Other solid tumours

Long-term efficacy results of a placebo-controlled phase III trial, including 773 cancer patients other than breast and prostate with skeletal metastases, showed that 4 mg zoledronic acid can significantly reduce the proportion of patients who experience at least one SRE (39% in zoledronic arm vs 48% with placebo, *p* = 0.039). Moreover, 4 mg zoledronic acid significantly delayed the median time to first SRE compared with placebo (236 vs 155 days, respectively, *p* = 0.009) and reduced the risk of SREs by 31% versus placebo in the overall trial population [39].

### Denosumab

Denosumab is a fully human, monoclonal, synthetic, IgG2 antibody that binds to RANKL, with high affinity and specificity, and inhibits formation, function, and survival of activated osteoclasts, bone destruction, and tumour growth.

Denosumab is recommended to be administered at a dose of 120 mg as a single subcutaneous (sc) injection once every 4 weeks taking supplemental calcium and vitamin D. It was approved by European Medicines Agency and Food and Drug Administration (FDA) for prevention of SREs in patients with solid tumours [40, 41]. It is recommended to start its use at the bone metastases diagnosis and should be continued indefinitely during the disease.

Denosumab toxicity profile consists mainly of hypocalcaemia and side effect, such as ONJ, occurs at similar low rates of zoledronic acid. However, acute-phase reactions and renal impaired are less frequent without the need for dose adjustment for renal disfunction or renal monitoring.

#### Breast cancer

A phase III study compared denosumab to zoledronic acid in patients with bone metastases from MBC [42]. A total of 2046 patients were randomized to receive, q4w, denosumab 120 mg sc plus placebo iv over 15 min (*n* = 1026) or placebo sc plus zoledronic acid 4 mg iv over 15 min (*n* = 1020). The primary objective was the time to first SRE with a statistical consideration of non-inferiority. Denosumab significantly delayed the time to first SRE compared with zoledronic by 18% (*p* = 0.01). Risk reduction in first and subsequent SREs consistently favoured denosumab also. Overall incidence of adverse events was similar between the two groups, including ONJ (*p* = 0.13). There were an increased incidence of acute-phase reactions in the zoledronic acid group and higher incidence of hypocalcaemia in the denosumab group.

In summary, denosumab is considered superior to zoledronic acid in delaying or preventing SREs in patients with breast cancer metastatic to bone with the convenience of a subcutaneous injection and no requirement for renal monitoring, and without differences in ONJ rates.

#### Prostate cancer

Denosumab (120 mg sc) was compared 1:1 to 4 mg iv zoledronic acid in a pivotal, double blind, placebo-controlled, phase III study in 1901 patients with bone metastases from RCPC, naïve to biphosphonates [43]. Time to first SRE, primary endpoint, was 20.7 months in denosumab group vs 17.1 months in ZA group (RR 0.82; CI 95% 0.71–0.95; *p* = 0.008). No differences in OS or time to progression were observed. ONJ was 2% in denosumab group and 1% in zoledronic group (*p* = 0.09). Adverse events related with acute-phase reactions were less common with denosumab (8 vs 18%; *p* < 0.0001) and doses changes were no required in denosumab patients if renal impairment.

In summary, and in agreement with recent guidelines for the treatment of bone metastases [44], it is recommended the administration of zoledronic acid or denosumab to prevent or delay SREs in patients with bone metastases from RCPC. Denosumab has superior clinical benefits and better tolerance.

#### Lung cancer

Denosumab has been studied in 1776 patients with skeletal metastases from a solid tumours and multiple myeloma other than breast and prostate cancer in a phase III trial comparing denosumab vs zoledronic acid. Denosumab improved the time to first SRE from 16.3 to 20.6 months (HR 0.84; *p* = 0.0007) achieving the primary endpoint of non-inferiority. Considering the effect of denosumab relative to zoledronic acid by tumour stratification factors for other solid tumours, and taking into account only non-small cell lung cancer (NSCLC) patients (*n* = 702), resulted in an HR of 0.84, *p* = 0.20 [41]. In an exploratory analysis performed for OS, denosumab was associated with improved median OS vs zoledronic acid in 811 patients with any lung cancer (8.9 vs 7.7 months, *p* = 0.01), in 702 patients with NSCLC (9.5 vs 8.0 months, *p* = 0.01), and 8.6 vs 6.4 months (*p* = 0.035) in patients with squamous cell carcinoma; however, without statistically significant differences for adenocarcinoma patients (HR 0.81; *p* = 0.36) and small cell lung cancer patients (HR 0.80; *p* = 0.075) [45].

Preclinical data [46] and these hypothesis-generating outcomes warrant further investigation: two prospective studies in lung cancer are ongoing to elucidate the therapeutic potential of denosumab beyond SRE prevention.

#### Other solid tumours

In the phase III trial, comparing denosumab with zoledronic acid considering the effect by tumour stratification factors for patients with bone metastases from only solid tumours other than MBC and prostate cancer, excluding NSCLC and multiple myeloma (a total of 904 patients) resulted in an improvement of the time to first SRE (HR 0.79; *p* = 0.04) [41]. In an ad hoc analysis of this study excluding only multiple myeloma (a total of 1597 patients), superiority of denosumab was observed delaying time to first on-study SREs (HR 0.81; *p* = 0.017), and time to first-and-subsequent SREs (RR 0.85, 95% CI, 0.72–1.00) and also prevented pain progression [47].

In summary, denosumab is more effective than zoledronic acid in delaying or preventing skeletal morbidity in patients with bone metastases from other solid tumours.

### Radiopharmaceuticals (including radium 223)

Radiopharmaceuticals are other class of bone-targeted therapies. Due to its similarity with the calcium, these agents are up-taken in sites with osteoblastic activity, where they destroy surrounding tissues because of their radioactive activity.

Radiopharmaceuticals are classified depending on the particles they emit. Strontium 89 and Samarium 109 are β-emitter particles with high tissue penetrance, leading risk of haematological toxicity, mainly thrombocytopenia [48]. These two agents did not demonstrated improved OS in phase III clinical trials, but FDA approved them, since a palliative relief of pain was achieved [49, 50].

Radium dichloride 223 emits alpha particles, with high energy but low penetration (<100 μm or a range of 2–10 cells of diameter), and acts in the microenvironment of bone metastases [51]. The recommended dosing is 50 kBq/kg (1.35 microcuries/kg) in 1-min bolus iv administration every 4 weeks, 6 doses. Due to the potential haematological toxicity of radium 233, adequate bone-marrow function parameters—haemoglobin ≥10 g/dL, absolute neutrophil count ≥1.5 × 10^9^/L, and platelets >100 × 10^9^/L—are required.

The randomized, double blind, ALSYMPCA [52] trial included 921 patients with bone metastases from RCPC, without visceral disease, after progression to docetaxel or unfit for this agent. Patients were assigned 2:1 to radium-223 plus best standard care (BSC) vs placebo + BSC, which included extern radiotherapy (RT), BPs, corticoids, antiandrogens, estroogens, estramustine, or ketoconazol. In a preplanned interim analysis, overall survival, primary endpoint, was superior with radium-223 [14 vs 11.2 months, (HR 0.70; 95% CI 0.55–0.88; *p* = 0.002). In a recent update, median OS was 14.9 and 11.3 months with radium-223 and placebo, respectively [53]. Time to first SRE was also longer with radium-223 vs placebo (median, 15.6 vs 9.8 months; HR 0.66; 95% CI 0.52–0,83; *p* < 0.001). Radium 223 decreased requirements of external RT for bone pain (HR 0.67, 95% CI 0.53–0.85) and risk of spinal cord compression (HR 0.52, 95% CI 0.53–0.85). This benefit was independent of previous docetaxel or concomitant bisphosphonates treatment. Hematologic toxicity in patients treated with chemotherapy after radium-223 was not higher than in the placebo group. Other side effects were similar in both groups. QOL tests (FACT-P) showed a significant improvement in the radium-223 arm versus placebo arm. There are ongoing clinical trials combining radium 223 with docetaxel and with other active compounds for RCPC.

In summary, radium-223 is the first therapy against the bone microenvironment that increases overall survival in RCPC patients. As a result, radium-223 was approved for patients with CRPC with symptomatic metastases and without visceral disease by FDA in May 2013.

## Other treatment options for bone metastases

The proper management of bone metastases requires multidisciplinary approach by radiologists, radiation oncologists, medical oncologists, surgeons, pain medicine specialists, and palliative care professionals. The goals of palliative treatment are pain relief, preservation of function, and maintenance of skeletal integrity. Early intervention may be useful in maintaining QOL and minimizing side effects of analgesic medication.

### External beam radiotherapy

Radiation therapy is the treatment of choice for palliation of localized bone pain. It is effective in a majority of patients, although a transient worsening of pain may occur in some cases (30–40%), typically in the first few days after RT, but this flare generally lasts 1 or 2 days [54]. Dexamethasone may reduce the frequency of pain flare.

Multiple prospective randomized trials have shown pain relief equivalency for dosing schema including 30 Gy in ten fractions, 24 Gy in six fractions, 20 Gy in five fractions, and a single 8 Gy fraction for patients with previously un-irradiated painful bone metastases [55].

Reirradiation may be necessary for patients if the initial treatment fails or there is a subsequent relapse after an initial response. There are limited data on the optimal schedule and dose for reirradiation [56–58].

### Stereotactic body radiation therapy

A newer technology to treat bone metastases is stereotactic body radiotherapy (SBRT). In selected cases with small-volume skeletal metastasis, limited metastatic tumour burden, and good performance status, SBRT provides a way to deliver a radical treatment. Current published results suggest that a single-fraction stereotactic radiosurgery at up to 20 Gy can be used for relief of acute bone pain, even for radio-resistant tumour types, such as melanoma and renal cell carcinoma. The optimal dose schedule for specific tumour types is not known [59].

Traditionally, RT to the vertebral body is limited by the tolerance of the spinal cord and reirradiation of the same vertebra is discouraged due to the potential for spinal cord injury. However, two small series reported good results with salvage SBRT for previously irradiated spinal metastases [60, 61].

### Orthopaedic surgery

A preoperative evaluation and systemic staging is mandatory. The aims are to delineate the osseous and soft tissue extent of the lesion and its relationship to adjacent structures, determine the overall skeletal involvement by tumour, detect any other metastases that may require concomitant treatment, and asses the patient´s overall prognosis. Pathological fractures are not an emergency, and proper workup is needed before any intervention.

Operative intervention for metastatic disease is generally a palliative procedure. Complete and impending pathologic fractures should be treated with durable fixation or reconstruction depending on the patient’s expected survival. Resections are done in selected cases, particularly for solitary metastases.

### Surgery for spinal metastases

Surgical treatment may be indicated for patients with spinal metastases that are causing instability or spinal cord compression. An unstable spine should be stabilized either by surgery with fixation or by percutaneous vertebral repair. Vertebroplasty and Kyphoplasty are reserved for patients with symptomatic vertebral body fractures without epidural disease or retropulsion of bone fragments into the spinal cord. Although clinical experience is limited, ASTRO guidelines recommend that radiotherapy be used in conjunction with these procedures [62].

Spinal cord compression is a devastating complication. Standard treatment consists of corticosteroids and RT, with which only about 50% of patients are able to walk and few non-ambulatory patients ever walk again. The amount of surgery ranges from simple decompression to a bloc resection and fixation. Patients with solitary metastasis with favorable histology, such as MBC, are more often selected for the bloc resections in view of longer life and, therefore, need to maintain QOL.

Results with laminectomy plus radiation did not seem to differ from results with RT alone. Surgical treatment was largely abandoned when several retrospective studies and a small randomized trial did not show any benefit for laminectomy alone or in combination with RT. However, these non-randomized studies were subject to patient selection bias, heterogeneous tumour types, unclear inclusion criteria, and imprecise endpoints. To determine the value of surgery, a randomized trial comparing the efficacy of direct decompressive surgery plus postoperative RT vs RT alone was taken. It demonstrated that for patients with good performance status and a life expectancy of at least 3 months, direct decompressive surgery followed by postoperative RT is clearly superior to RT alone in terms of maintaining deambulation and need for corticosteroids and opioid analgesics was significantly reduced in the surgical group [63].

#### Long bones

The usual surgical options vary from internal fixation with a nail or plate with or without cement and endoprosthetic arthroplasty. The exact choice depends on the location, amount of bone loss, and responsiveness of lesion to systemic therapy. As a general rule, nails are preferred in the lower limbs and plates in the upper limb. Wherever possible, the maximum length of the bone is stabilized to minimize a future fracture at another location [64].

#### Pelvic and periacetabular defects

Surgery is rarely required for pathologic fractures of the pelvis, except for those involving the acetabulum. Most pathologic acetabular fractures are addressed with a complex acetabular prosthetic arthroplasty reconstruction that distributes the stresses away from the diseased bone and into the intact bone of the superior ilium when intact. Increasingly, painful osteolytic defects around pelvis and acetabulum have begun to be treated with percutaneous procedures, including cementoplasty, radiofrequency ablation, cryoablation, and focused ultrasound.

In summary, for patients with a single or limited number of areas of painful bone metastases, external RT is indicated. Using a single fraction of 8 Gy to the involved area may provide equal palliation with improved patient convenience and cost effective compared with fractioned schedules. SBRT may be particularly useful in selected cases with radio-resistant tumours, spinal cord compression, where there is a significant concern about normal tissue toxicity and for those who need retreatment for previously spinal metastases.

Surgical decompression and stabilization plus postoperative RT should be considered for selected patients with single level spinal cord compression or spinal instability, unless the patients have too short of an anticipated life expectancy. Kyphoplasty and vertebroplasty may be useful for the treatment of lytic osteoclastic spine metastases or in cases of spinal instability, where surgery is not feasible or indicated; they do not obviate the need for external beam RT. Radiotherapy is indicated for most patients after fixation of a complete or impending pathologic fracture of any bone.

## Prevention of cancer treatment-induced bone loss

In the adjuvant setting, cancer treatment-induced bone loss is a frequent cause of morbidity, and prevention and treatment of this condition with BPs and denosumab are also well established. Besides postmenopausal patients, several studies, including two large studies by the Austrian Breast and Colorectal Cancer Study Group (ABCSG) and the Cancer and Leukemia Group B (CALGB), have shown an increase in bone mineral density in premenopausal women.

## Adjuvant bone-targeted therapy in breast cancer

Clinical trials of BPs as adjuvant therapy for breast cancer have had mixed results. Clodronate, an oral first-generation bisphosphonate, showed a DFS benefit vs placebo in one large randomized trial [65], but not in another [66]. An early trial of zoledronic acid added to adjuvant aromatase inhibitor therapy for postmenopausal women to prevent bone loss, showed a non-significant improvement in DFS [67], a secondary endpoint. Larger trials comparing zoledronic acid to no therapy in postmenopausal women [68], or in premenopausal women made menopausal with gonadotropin-releasing hormone agonists [69], showed significant DFS benefits, but no benefit was seen in a large randomized trial of both premenopausal and postmenopausal women [70].

The Early Breast Cancer Trialists’ Collaborative Group [71] presented a meta-analysis comprised of individual patient data derived from randomized adjuvant bisphosphonate trials in breast cancer done over the past 20 years. The analysis received data on 18 766 women (18 206 in randomized trials of 2–5 years of adjuvant BPs vs control), with a median follow-up of 5.6 years, 3453 first recurrences, and 2106 deaths. For all patients, there were borderline significant reductions with the addition of bisphosphonates at 10 years for distant recurrence (20.4 vs 21.8%, *p* = 0.03), bone recurrence (7.8 vs 9.0%, *p* = 0.004), breast cancer mortality (16.6 vs 18.4%, *p* = 0.04), and all-cause mortality (20.8 vs 22.3%, 2*p* = 0.06).

In postmenopausal women, there were highly significant reductions with the addition of bisphosphonates at 10 years for bone recurrence (6.6 vs 8.8%, *p* = 0.0002) and for breast cancer mortality (14.7 vs 18.0%, *p* = 0.002). This benefit was independent of the type and schedule of administration of BP, the oestrogen receptor status of the primary tumour, the presence of axillary lymph node involvement, and the use of concomitant systemic chemotherapy. There was no reduction in the incidence of contralateral breast cancer or in the risk of metastasis to non‐osseous sites. In the 13,341 women with available fracture data, BPs reduced the risk of fracture from 7.3 to 6.3% (*p* = 0.02).

The absolute reduction in the risk of breast cancer death at 10 years with the use of bisphosphonates in postmenopausal women (3.3%) is similar to the benefit seen with anthracycline polychemotherapy vs non-anthracycline polychemotherapy [72]. The publication of the latest Oxford overview of prospective trials is being awaited; at the presentation of the results, a 34% relative reduction of bone metastases and a 17% improvement in OS were demonstrated in the subgroup of postmenopausal patients.

These results on breast cancer treatment could lead to widespread adoption of bisphosphonates as a standard of care for the adjuvant therapy of early stage breast cancer in postmenopausal women.

### Should all postmenopausal women with early stage breast cancer receive adjuvant bisphosphonates?

Bisphosphonate action seems to be bone centric, and as a result, BPs do not appear to prevent contralateral breast cancer, locoregional disease, or metastases to non-osseous sites. One study indicating that overexpression of lysyl oxidase by primary breast cancers can lead to increased bone turnover in the bone micro-environmental niche and increased establishment of bone metastases is intriguing, and suggests a potential biomarker for response [73]. Increased bone turnover after menopause might also explain the preferential benefit of BPs in reducing bone recurrence in postmenopausal women. It will be interesting to determine if osteoprotogerin analogues, such as denosumab, provide DFS benefits similar to BPs in ongoing clinical trials, such as ABCSG-18 [74], in which denosumab significantly reduced fracture rates in postmenopausal women receiving aromatase inhibitors.

This apparent success in altering the natural history of breast cancer by modifying the tumour microenvironment lends great credence to investigational efforts in breast cancer and other cancers attempting to understand and modify tumour–host micro-environmental interactions.

### Other adjuvant studies

The study D-CARE (ClinicalTrials.gov Identifier: NCT01077154) is a prospective, randomized, ongoing phase III trial comparing denosumab vs placebo (in addition to standard adjuvant therapy) in 4509 patients with operable breast cancer. The primary end-point of the trial is bone metastases-free survival.

## Recommendations

Zoledronic acid is the most active biphosphonate to prevent morbidity from bone metastases in patients with breast, castration-resistant prostate, lung, and other solid cancers. It is administered at a dose of 4 mg iv every 3 or 4 weeks when bone metastases are detected, even the patient is asymptomatic, and should be continued during the disease. In metastatic breast cancer, a dosis of 4 mg every 12 weeks may be considered.

Denosumab is more effective and more convenience than zoledronic acid in delaying or preventing skeletal morbidity in patients with bone metastases from several types of cancers. Denosumab does not need dosing adjustment in case of renal impairment.

Radium-223 increases overall survival in castration-resistant prostate cancer and is a new bone-targeted agent for this disease.

Multidisciplinary approach is essential. Radiation and surgery should be considered in all patients, and indicated when appropriate.
